# The Effect of *Zataria multiflora* on Th1/Th2 and Th17/T Regulatory in a Mouse Model of Allergic Asthma

**DOI:** 10.3389/fphar.2017.00458

**Published:** 2017-08-07

**Authors:** Majid Kianmehr, Dariush Haghmorad, Reza Nosratabadi, Abdolrahim Rezaei, Azam Alavinezhad, Mohammad Hossein Boskabady

**Affiliations:** ^1^Neurogeneeic Inflammation Research Centre, Mashhad University of Medical Sciences Mashhad, Iran; ^2^Department of Physiology, School of Medicine, Mashhad University of Medical Sciences Mashhad, Iran; ^3^Department of Immunology, Faculty of Medicine, Semnan University of Medical Sciences Semnan, Iran; ^4^Immunology of Infectious Diseases Research Center, Rafsanjan University of Medical Sciences Rafsanjan, Iran; ^5^Department of Immunology, Faculty of Medicine, Rafsanjan University of Medical Sciences Rafsanjan, Iran; ^6^Inflammation and Inflammatory Diseases Research Center, Mashhad University of Medical Sciences Mashhad, Iran

**Keywords:** animal model of asthma, flow cytometry, mice, real time PCR, *Zataria multiflora*

## Abstract

Asthma is a chronic inflammatory disease with no definite treatment and more research is needed to overcome this condition. The aim of this study was to investigate the effect of the extract of *Zataria multiflora* (*Z. multiflora*) as a medicinal plant on cytokine genes expression in an experimental mouse model of asthma. Adult mice were randomly divided into the following groups: control (C), untreated asthma (A), asthmatic groups treated with dexamethasone (D) and *Z. multiflora* extract (200, 400, and 800 μg/mL; Z1, Z2, and Z3, respectively), (for groups C, A, and D *n* = 5 and for groups Z1, Z2, and Z3 *n* = 6). For induction of the mouse model of asthma, animals were sensitized with intraperitoneal injection and inhalation of ovalbumin (OVA). The number of T helper (Th) subtype cells (using flow cytometry) and the levels of IFN-γ, FOXP3, IL-4, TGF-β, IL-17 gene expression (by real time PCR) were assessed in mice splenocytes. The observed changes in spleen cells of group A compared to group C were increased number of Th_2_ and Th_17_ cells, enhancement of gene expression of IL-4, IL-17, and TGF-β (*p* < 0.001 for all cases), reduction of Th_1_ cells and Th_1_/Th_2_ ratio (*p* < 0.001 for both cases) and decrease in gene expression of IFN-γ, FOXP_3_ and IFN-γ/IL-4 ratio (*p* < 0.01 for IFN-γ and *p* < 0.001 for other cases). The observed changes in spleen cells of treated compared to untreated A group were enhancement of Treg cells and Th_1_/Th_2_ ratio (*p* < 0.001 for both cases), increase in IFN-γ (*p* < 0.05) and FOXP_3_ (*p* < 0.001) gene expression and IFN-γ/IL-4 ratio (*p* < 0.01) as well as reduction of Th_2_ and Th_17_ cells (*p* < 0.01 to *p* < 0.001), decrease gene expression of IL-4, IL-17, and TGF-β (*p* < 0.05 to *p* < 0.001). The findings showed that the extract of *Z. multiflora* decreased pro-inflammatory cytokines in asthma (IL-4 and IL-17 and TGF-β) but increased anti-inflammatory cytokines (IFN-γ) gene expression and the number of Treg (FOXP3) in splenocytes of asthmatic mice which may indicate the specific therapeutic effect of the plant extract in allergy, autoimmunity, and infectious diseases via potentiating Th_1_ and suppressing Th2 and Th_17_ cells.

## Introduction

There are a growing use of herbal medicines and natural products, especially in recent years. Currently, 25% of existing drugs are made from herbal sources and 10% of medications are produced from microbial sources (Demain and Sanchez, 2009; Gurnani et al., 2014). *Zataria multiflora* (*Z. multiflora*) is native to Southwestern Asia such as Iran, Afghanistan, Pakistan, and Kashmir (Manikandan et al., 2012). This plant contains compounds such as thymol and carvacrol which are likely to be responsible for its pharmacological effects. The amount of thymol in fresh plant was reported as 73.21% while in dried plant the amount of carvacrol is 62.87% (Saleem et al., 2004). Various pharmacological and therapeutic effects were reported for *Z. multiflora* including effect on gastrointestinal disorders such as reduction of colon inflammation, and duodenal ulcer in inflammatory bowel disease (Minaiyan et al., 2005; Nakhai et al., 2007), dose-dependent anti-nociceptive activity (Hosseinzadeh et al., 2000), anti-microbial mainly on streptococci and *E. coli* (Owlia et al., 2004; Rahmani et al., 2012a,b), anti-fungal activity against *Saprolegnia parasitica* (Khosravi et al., 2012; Mahammadi purfard and Kavoosi, 2012), anti-oxidant (Babaie et al., 2007; Karimian et al., 2012; Boskabady and Mahtaj, 2015), and anti-inflammatory properties against acute and chronic inflammation, and lung inflammation (Hosseinzadeh et al., 2000; Jaffary et al., 2000; Boskabady et al., 2013, 2014b). The immuno-modulatory effects of the plant such as increased IFN-γ and IFN-γ/IL-4 ratio (Th_1_/Th_2_ balance) as well as decreased IL-4 were also demonstrated (Boskabady et al., 2013). In addition, several pharmacological effects of *Z. multiflora* were shown on respiratory system including stimulation of β2 adrenoceptors, inhibition of muscarinic (Boskabady et al., 2010, 2012b,a; Jafari et al., 2011) and histamine (H_1_) receptors (Boskabady et al., 2012b). Reduction of tracheal responsiveness (Gholami Mahtaj et al., 2015) and inflammatory mediators (Boskabady et al., 2013; Boskabady and Mahtaj, 2015) as well as improvement of lung pathological changes (Gholami Mahtaj et al., 2015) in sensitized guinea pigs due to treatment with the plant extract were also demonstrated. In addition, the preventive effects of *Z. multiflora* on emphysema and pathological changes of the lung and systemic inflammation in animal models of COPD (Gholami Mahtaj et al., 2015) were documented. The effects of the extract of *Z. multiflora* on coughs due to colds, bronchitis, disorders of the oral cavity (Mozaffarian, 1996), respiratory disorders of chemical war victims (Mostafavi and Shasavari, 2005) and its antitussive effect (Afzali et al., 2003) were also shown. The plant also traditionally used as an antibacterial agent for oral hygiene in Iran (Avicenna, 1985).

Asthma is mainly characterized by: (1) Intermittent and reversible obstruction of the airways which leads to recurrent attacks of wheezing, shortness of breath and cough (2) Bronchial hyper-responsiveness (BHR), which is the narrowing of the airways as a result of small amounts of broncho constrictor agents such as histamine or cholinergic agonists and (3) Airway inflammation. Cytokines play a key role in organizing the chronic inflammation of asthma. Increased number of CD4+ Th cells and subsequent increased secretion of IL-4, IL-5, IL-9, and IL-13 from Th_2_ cells, were shown in the airways of asthmatic patients (Barnes et al., 1998). Therefore, Th2 cells can induce allergic inflammatory diseases (Salmon et al., 1999; Babayigit et al., 2009). The above-mentioned cytokines stimulate B cells to induce immunoglobulin E (IgE) secretion, eosinophilic inflammation and mast cell proliferation. IFN-γ is the predominant cytokine produced by Th_1_ and its level is reduced in individuals with asthma (Renauld, 2001; Mehta and Mahajan, 2006; Barnes, 2008). Th_1_ can inhibit Th_2_ activity, and enhancement of Th1 activity could be a possible asthma therapy (Boskabady et al., 2013). Therefore, changes in IFN-γ/IL-4 cytokines ratio (Th_1_/Th_2_ balance) toward IL-4 (Th_2_) may cause allergic and atopic diseases such as atopic dermatitis, anaphylactic shock, allergic rhinitis, and asthma.

The regulatory T cells (Treg) activity is regulated by a specific transcription factor namely forkhead/winged helix (FOXP_3_) transcription factor. Treg cells normally suppress other Th cells by releasing TGF-β and IL-10 and the function of Treg may be impaired in asthma (Fontenot et al., 2003; Hori et al., 2003; Khattri et al., 2003; Barnes, 2008). Th_17_ cells can cause neutrophilic inflammation, airway remodeling and irreversible airway obstruction via secretion of IL-17. IL-17 also inhibits FOXP_3_ expression (Bullens et al., 2006) and has an important role in the progress of autoimmune disorders (Annunziato et al., 2008).

In this study, the preventive effect of *Z. multiflora* administered during the sensitization period was examined on subsets of T cells determination by flow cytometry and their cytokine gene expression in splenocytes of experimentally-induced asthma in BALB/c.

## Materials and methods

### Animals and groups

BALB/c mice weighted 20–25 g and age of 6–8 weeks were used in this study. The animals were housed in cages under standard temperature (20 ± 2°C), humidity (55 ± 5%) and light (12 h: 12 h light: dark) conditions with food and water were available *ad libitum*, in the animal house of School of Medicine, Mashhad University of Medical Sciences, Mashhad, Iran.

Animals were randomly divided into the following groups:

(1) Non-asthmatic control animals (group C).
(2) Untreated asthmatic animals (group A).
(3-5) Asthmatic animals treated with *Z. multiflora* extract (200, 400, and 800 μg/mL) added to animals' drinking water (groups Z1, Z2, and Z3, respectively) during sensitization period. Each mouse drank an average of 4 ml drinking water per day and there was no significant difference in the amount of used drinking water among groups.
(6) Asthmatic group treated with dexamethasone (1 mg/kg/day; Sigma Chemicals, LTD.) from day 34 to 40 of sensitization period (group D). For groups C, A, and D, there were 5 animals in each group (*n* = 5) while in groups Z1, Z2, and Z3, there were 6 animals in each groups (*n* = 6).

### Induction of the mouse model of asthma and extraction of splenocytes

**Figure 1:**
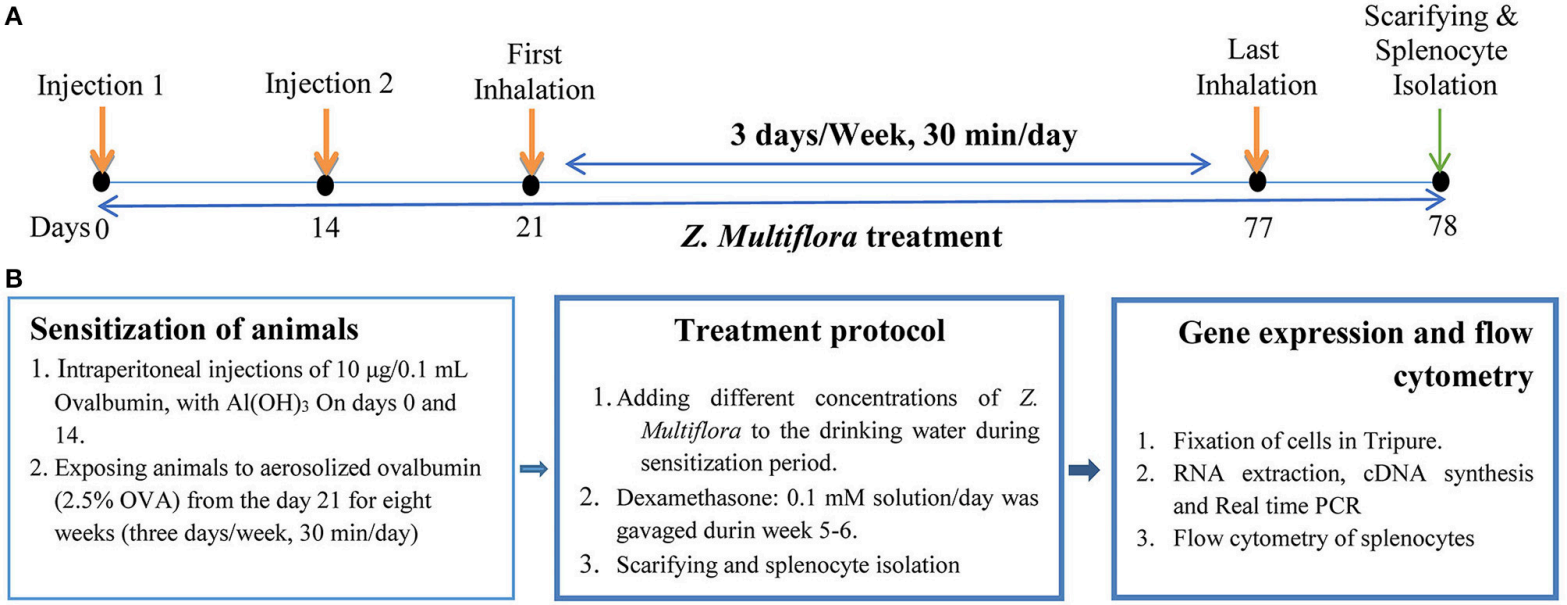
The schematic time-course of inducing animal model of asthma, treatment, spleocyte isolation, flow cytometry and gene expression. **(A)** Induction of experimental animal model of asthma (sensitization) in mice. In control animals saline was administrated instead of OVA. **(B)** Treatment of animals with dexamethasone and three concentrations of *Z. multiflora*. In control and sensitized groups: animals were given drinking water alone. In group D: dexamethasone (0.1 mmol) was gavaged from the beginning of week 5 for 1 week. In groups Z1, Z2, and Z3: animals were given drinking water containing extract of *Z. multiflora* at three concentrations of 200, 400, and 800 μg/mL.

On days 0 and 14 of the experiment, mice were sensitized by intraperitoneal (i.p) injections of 10 μg/0.1 mL chicken egg albumin (Ovalbumin or OVA, grade V, 98% pure; Sigma, St. Louis, MO, USA) together with Al(OH)_3_. From the day 21 of the study, animals were exposed to aerosolized ovalbumin (2.5% OVA) for 8 weeks (30 min/day, 3 days/week; Temelkovski et al., 1998; Babayigit et al., 2009; Figures 1A,B). During experimental period, non-sensitized animals (control group) received injected and aerosolized saline instead of OVA. To evaluate the sensitivity of the animal, DTH (delayed-type hypersensitivity) test was carried out. For this purpose, 0.1 mg OVA (up to 10-fold diluted) was injected subcutaneously. Increased skin thickness, as well as inflamed and redness of the skin 1 day after injection indicated animal sensitization.

One day after the last inhalation (day 78) animals were sacrificed, spleen was isolated and its lymphocytes were extracted and cultured. The study protocol was approved by the Ethics Committee, Mashhad University of Medical Sciences and all experiments were conducted in accordance with the National Institute of Health guide for the care and use of laboratory animals (NIH Publications No. 80-23).

### Plant and extracts

*Z. multiflora* was collected from mountains between Tabas and Yazd, in central Iran. The plant was identified by botanists and kept in the herbarium of Ferdowsi University of Mashhad with herbarium number: 35314. For preparation of hydro-ethanolic extract, 100 g of dried plant was soaked with 875 mL of 50% ethanol for 72 h at room temperature. The extract was then passed through the filter paper, the solvent was removed under reduced pressure and the dried extract was kept in refrigerator. The yield extract was 33.2 g. Three concentrations (200, 400, and 800 μg/mL) of the extract were then used for treatment groups.

### HPLC finger print of *Z. multiflora* extract

**Figure 2:**
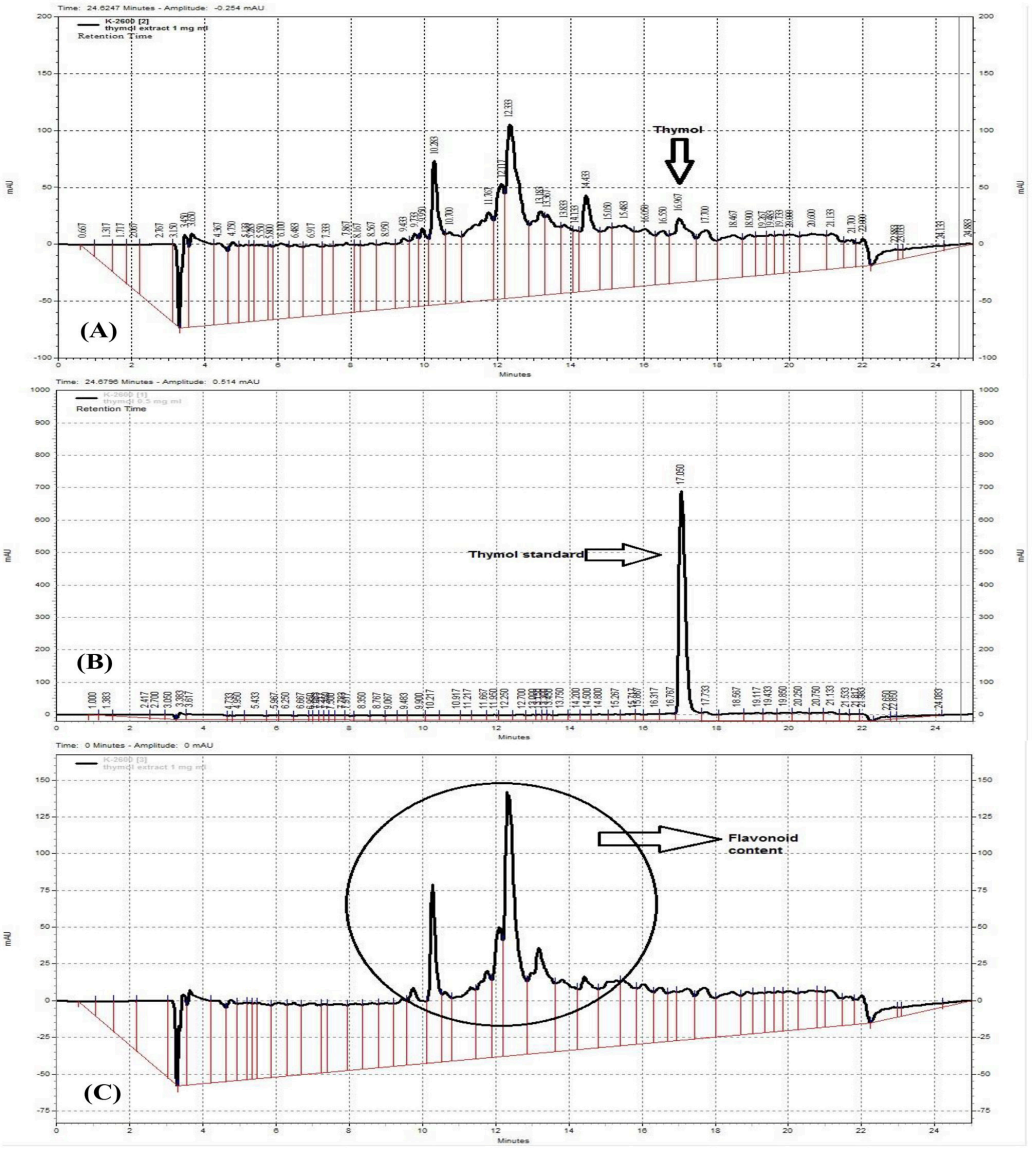
RP-HPLC fingerprinting analysis of *Z. multiflora* extract **(A,C)** at 280 and 320 nm **(C)**, thymol standard curve **(B)**. The presence of flavonoids was confirmed at the wavelength 320 nm which is specific for flavonoids and on the basis of previous works.

The hydro-ethanolic extract of *Z. multiflora* was characterized using a fingerprint profile prepared by a reversed-phase high performance chromatography (RP-HPLC). RP-HPLC was carried out by a linear gradient mobile phase included 0–100% methanol with a 1 ml/min flow rate at RT. Sample was prepared by filtering through 0.45 μm pore size ultra-membrane filter followed by sonication for about 45 min. It was loaded on a C18 column (5 μm particle size, 250 × 4.6 mm) from Capital (Broxburn, UK) with 20 μl injection volume. HPLC-UV (multi-wavelengths) measurements were performed using a Knauer HPLC system (Berlin, Germany) equipped with UV detector K-2600 and K-1001 HPLC Pump. The HPLC system was supplied with Chromgate HPLC software, version 3.3 and analyses were monitored at 240, 280, and 320 nm (Figure 2).

### Flow cytometry

To determine different subsets of regulatory T cells, single-cell suspension prepared from mice spleen (1 × 10^6^ of cells) was poured in flow tube and surface stained with CD4 and CD25 and anti-mouse FoxP3^+^ according to eBioscience mouse regulatory T cell staining kit's guidelines (BD, eBioscience, USA). Spleen cells were suspended in the presence of PMA (Phorbol 12-myristate 13-acetate, Sigma), ionomycin (Sigma) and Brefeldyn for 4 h to evaluate Th_1_, Th_2_, and Th_17_ cells. According to kit protocol, for the entry of IFN-γ, IL-4, and IL-17 antibodies (Anti-Mouse IFN-γ, IL-4, and IL-17, PE eBioscience, USA) cells membrane was permeabilized by permeabilization buffer and fixed by fixation buffer. Then, all samples were analyzed using a BD FACSCalibur flow cytometer and the results were analyzed using Flowjo Software.

### Real-time PCR analysis

The RNA extracted from splenocytes (5 × 10^6^ cells) was mixed with 1 ml of Tripure Isolation Reagents (Roche Applied Science, Germany), and cDNA was prepared with cDNA Synthesis Kit (Fermentas, Germany) according to instructions, for quantitative real-time analysis.

Primers and quantitation probes for β2- Microglobulin (β2M), Interferon gamma (IFN-γ), IL-4, IL-17, FOXP_3_ and Transforming growth factor beta (TGF-β), were designed using the Beacon Designer software Version 7.9. The primers and probe are mentioned in Table 1.

**Table 1:** Sequences of primers used for real-time PCR.

**Gene name**	**Sence/antisence primers-probs**
1. β2-Microglobin	5-GCCGAACATACTGAACTGCTAC-3 (forward)
	5-CTTGCTGAAGGACATATCTGACATC-3 (reverse)
	5-AACACAGTTCCACCCGCCTCACATTGA-3 (probe)
2. IFN-γ	5-GTATTGCCAAGTTTGAGGTCAAC-3 (forward)
	5-GCTTCCTGAGGCTGGATTC-3 (reverse)
	5-CCACAGGTCCAGCGCCAAGCATTCAA-3 (probe)
3. IL-4	5-TCCTCACAGCAACGAAGAAC-3 (forward)
	5-CAAGCATGGAGTTTTCCCATG-3 (reverse)
	5-AGCACCTTGGAAGCCCTACAGACGAGC-3 (probe)
4. IL-17	5-CAGACTACCTCAACCGTTCC-3 (forward)
	5-TTCCCTCCGCATTGACAC-3 (reverse)
	5-ACTCTCCACCGCAATGAAGACCCTGA-3 (probe)
5. TGF-β	5-CCTGGATACCAACTATTGCTTCAG-3 (forward)
	5-CAGACAGAAGTTGGCATGGTAG-3 (reverse)
	5-TCCACTTCCAACCCAGGTCCTTCCT-3 (probe)
6. FOXP3	5-GGTACACCCAGGAAAGACAG-3 (forward)
	5-GCTTGGCAGTGCTTGAGA-3 (reverse)
	5-TGGCTCCTCGAAGACCTTCTCACAACC-3 (probe)

Real-time PCR was performed using TaqMan PCR Mastermix and pre-formulated primers for mentioned genes and β2M by Rotor Gene Q Real-Time PCR machine (Corbett Research, Australia). The results were analyzed by the comparative threshold cycle method and normalized by β2M as an internal control with Rotor Gene 6000 software (Corbett Research, Australia). Using the below equation, the fold change in expression of gene of interest for each group was calculated: mean of gene of interest normalized. Index of test group/mean of gene of interest normalized index of healthy controls.

### Statistical analysis

Data were expressed as mean ± SEM. Comparison between groups was performed using one way ANOVA with Tukey Kramer *post-hoc* tests. A *P*-value less than 0.05 was considered as statistically significant. For data analysis, SPSS software (version 16) was used.

## Results

### Characteristics of *Z. multiflora* extract

The HPLC fingerprint shows 5 major peaks in retention times between 10 and 18 min. According to thymol standard curve (Figure 2B), the peak appeared at 16.967 in Figure 2A is thymol. The quantification of thymol in the total extract of *Z. multiflora* was also carried out using HPLC. The total content of thymol was determined to be 1.6% w/w of the total extract (Figure 2A). According to previous studies, peaks of HPLC chromatogram appeared at 320 nm are ascribed to flavonoids (Figure 2C). These compounds include apigenin, luteolin and 6-hydroxyluteolin (Ali et al., 2000).

### Delayed-type hypersensitivity (DTH) results

All animals in the OVA-sensitized groups showed positive DTH test which confirm animal sensitization (induction of mice animal model of asthma).

### Flow cytometry results

#### Subsets of Th lymphocytes and Th_1_/Th_2_ balance in the spleen of asthmatic mice

**Figure 3:**
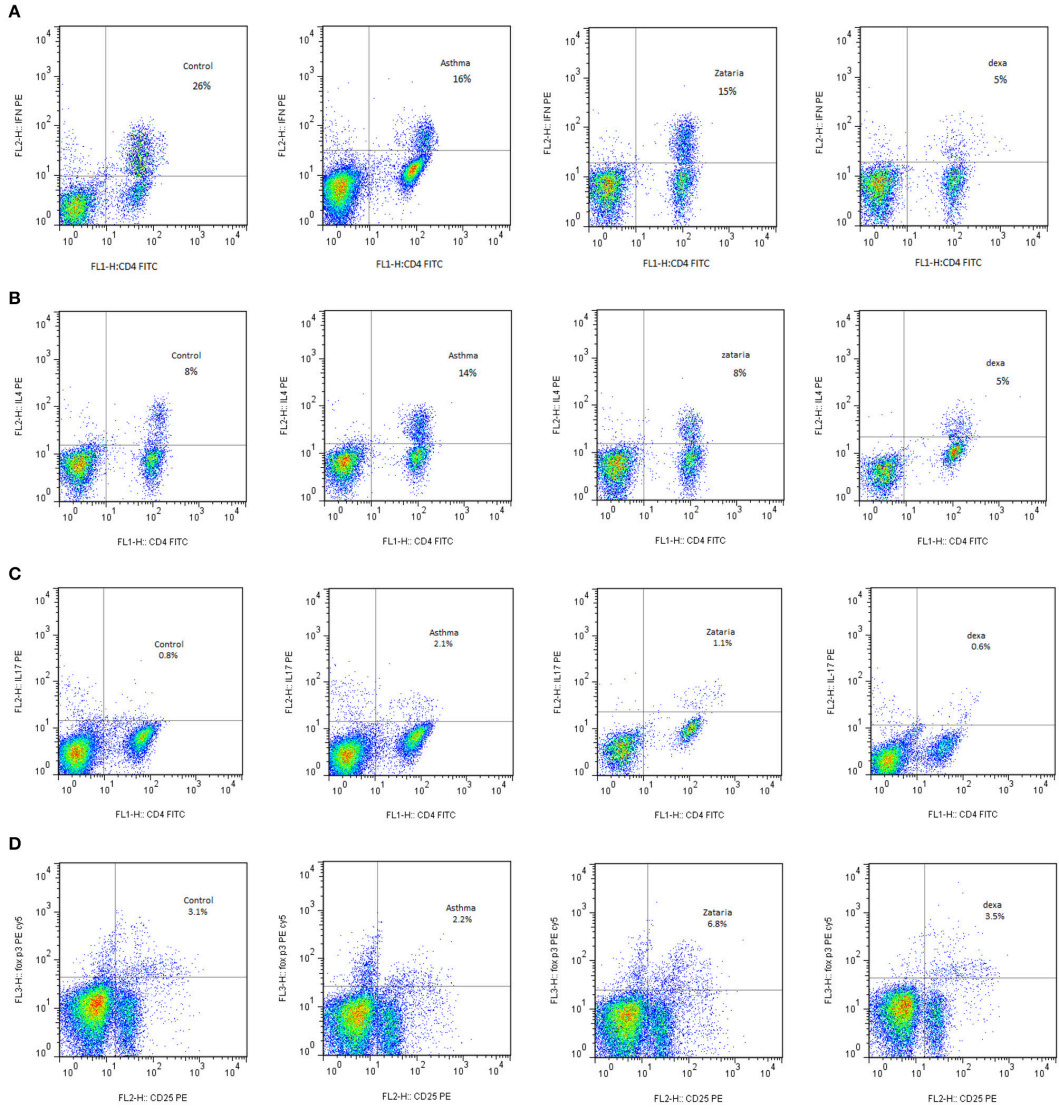
Flow cytometry of various T Cells sub types including; **(A)** In plot FSC and SSC, Th_1_ lymphocyte population between CD4 markers and IFN-γ were selected. The Th_1_ in the group treated with extracts in comparison with untreated group increased. **(B)** In plot FSC and SSC, Th_2_ lymphocyte population between the CD4 and IL-4 markers were selected. Th_2_ levels in treated groups with extract and dexamethasone compared with untreated group decreased. **(C)** In the plot FSC and SSC, Th_17_ lymphocyte population between the CD4 and IL-17 markers were selected. The Th_17_ in the treated groups with extract and dexamethasone compared with untreated group decreased. **(D)** Treg cells on the lymphocyte population were selected between Sec and CD4. Then this population on CD25 and FOXP3 was placed for determine the amount of these cells. Treg levels in groups treated with extracts and dexamethasone compared with untreated group increased.

**Figure 4:**
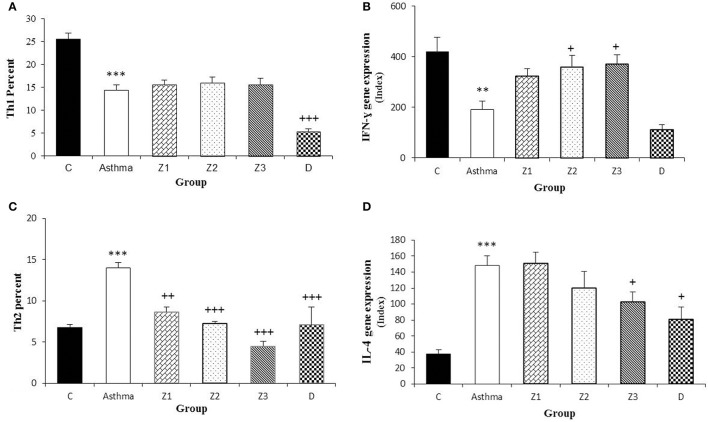
Percent of Th_1_ and Th_2_ cells **(A,C)** and values of IFN-γ and IL-4 gene expression **(B,D)** in splenocyte from control group (C, *n* = 5), Asthma group (A, *n* = 5) and A treated groups with the extract of *Z. multiflora* at concentrations 200, 400, and 800 μg/mL (groups Z1, Z2, and Z3, respectively, *n* = 6) and dexamethasone (D, *n* = 5). Data were presented as mean ± SEM. ^**^p < 0.01, ^***^p < 0.001, vs. control group. ^+^*p* < 0.05, ^++^*p* < 0.01, ^+++^*p* < 0.001, vs. asthma group. The statistical comparisons were made using ANOVA with Tukey–Kramer multiple *post-hoc* test.

**Figure 5:**
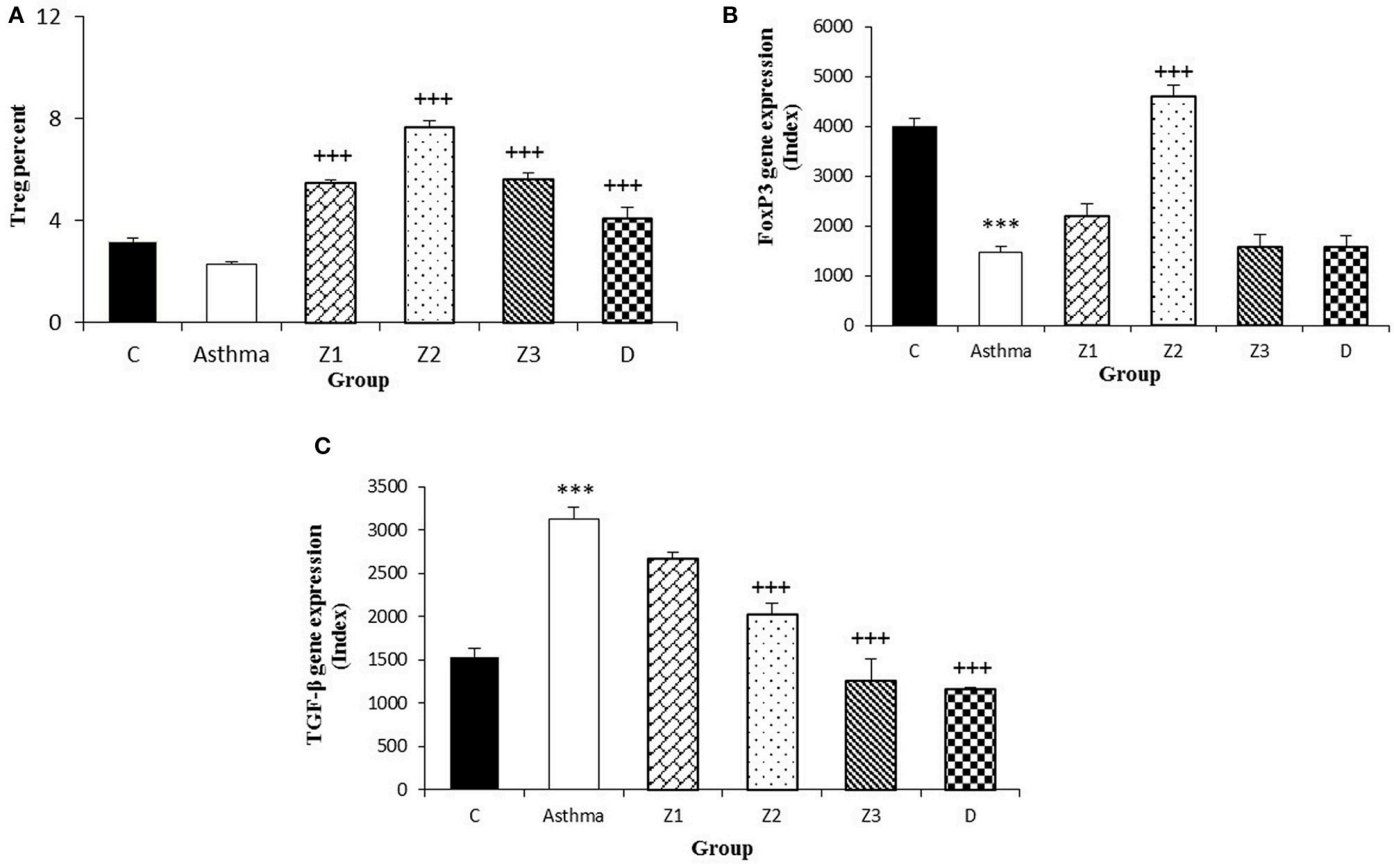
Percent of Treg cells **(A)**, values of FOXP3 **(B)**, and TGF-β **(C)** gene expression in splenocyte from control group (C, *n* = 5), Asthma group (A, *n* = 5), and A treated groups with the extract of *Z. multiflora* at concentrations 200, 400, and 800 μg/mL (groups Z1, Z2, and Z3, respectively, *n* = 6) and dexamethasone (D, *n* = 5). Data were presented as mean ± SEM. ^***^p < 0.001, vs. control group. ^+++^*p* < 0.001, vs. asthma group. The statistical comparisons were made using ANOVA with Tukey–Kramer multiple *post-hoc* test.

**Figure 6:**
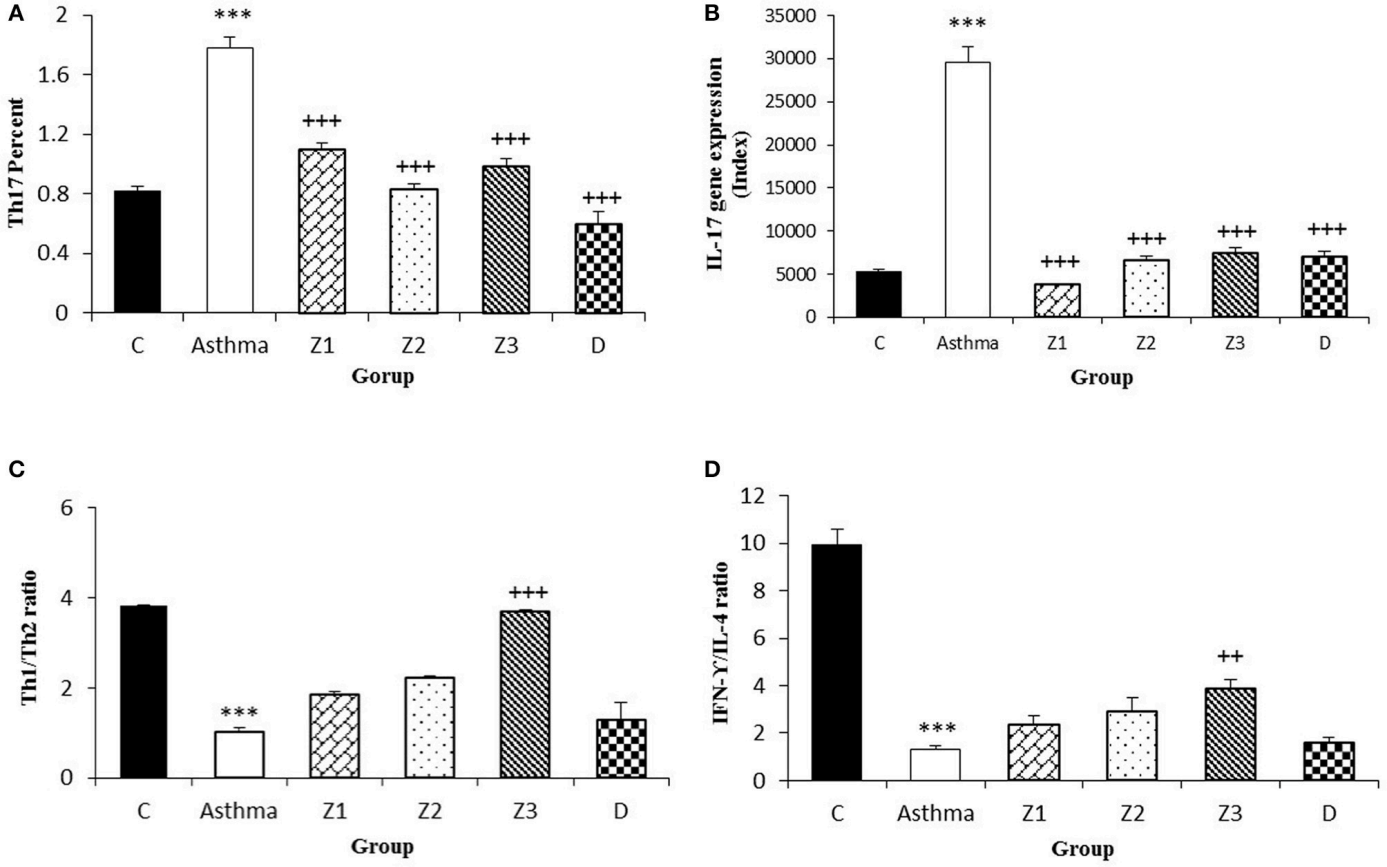
Percent of Th_17_ cells **(A)**, values of IL-17 gene expression **(B)**, Th_1_/Th_2_ ratio **(C)**, and values of IFN-γ/IL-4 ratio **(D)** in splenocyte from control group (C, *n* = 5), Asthma group (A, *n* = 5) and A treated groups with the extract of *Z. multiflora* at concentrations 200, 400, and 800 μg/mL (groups Z1, Z2, and Z3, respectively, *n* = 6) and dexamethasone (D, *n* = 5). Data were presented as mean ± SEM. ^***^p < 0.001, vs. control group. ^++^*p* < 0.01, ^+++^*p* < 0.001, vs. asthma group. The statistical comparisons were made using ANOVA with Tukey–Kramer multiple *post-hoc* test.

In asthma group, Th_2_ and Th_17_ cells were significantly increased but Th_1_ cells was significantly decreased (*p* < 0.001 for all cases), while Treg cells did not show significant change compared to the control group (Figures 3A,D, 4A,C, 5A). In addition, the ratio of Th_1_/Th_2_ in asthmatic group was significantly decreased compared to the control group (*p* < 0.001; Figure 6C).

#### Subsets of lymphocytes and Th_1_/Th_2_ balance in asthmatic animals treated with *Z. multiflora* extract and dexamethasone

Compared to untreated asthmatic group, Th_2_ and Th_17_ cells in asthmatic animals treated with all extract concentrations were significantly reduced (*p* < 0.01 for Th_2_ in Z1, and *p* < 0.001 for other cases; Figures 3B,D, 4C, 6A). Th_1_/Th_2_ ratio in asthmatic animals treated with high concentration of the extract (800 μg/ml) was significantly increased compared to untreated asthmatic group (*p* < 0.001; Figure 6C).

There was no significant difference in Th_1_ and Th_17_ among the asthmatic groups treated with different extract concentrations. The effects of treatment with high concentration of the extract (800 μg/ml) on Th_2_ were significantly higher than its low concentration (*p* < 0.05). The effect of high concentration of the extract on Th_1_/Th_2_ ratio was also significantly higher than its low concentration (*p* < 0.01; Table 2).

**Table 2:** The percent of T cells and values of IFN-γ, IL-4, TGF-β, FOXP3, and IL-17 gene expressions as well as IFN-γ/IL-4 ratio of splenocyte in asthmatic groups treated with the extract of *Z. multiflora* at concentrations 200, 400, and 800 μg/ml (groups Z1, Z2, and Z3, respectively) and dexamethasone (D, 0.1 mM).

**Variable**	**Z1**	**Z2**	**Z3**	**D**
Th_1_	15.66 ± 0.88^***^	16 ± 1.29^***^	15.66 ± 1.38^***^	5.4 ± 0.54
Th_2_	8.66 ± 0.55	7.25 ± 0.25	4.5 ± 0.56^#^	7.1 ± 2.12
Th_17_	1.1 ± 0.04^**^	0.83 ± 0.03	0.98 ± 0.05^*^	0.6 ± 0.08
Treg	5.45 ± 0.14^**^	7.66 ± 0.25^***^^,^^###^	5.63 ± 0.22^**^^,^^†††^	4.08 ± 0.45
Th_1_/Th_2_	1.85 ± 0.19	2.22 ± 0.19	3.68 ± 0.49^***^^,^^##^^,^^†^	1.27 ± 0.39
IFN-γ	323 ± 29.29^**^	359.9 ± 45.68^***^	371.5 ± 37.33^***^	112.4 ± 18.71
FOXP3	2,196 ± 254.1	4,599 ± 228.6^***^^,^^###^	1,565 ± 262.4^†††^	1,580 ± 223.2
IL-4	150.7 ± 13.97^*^	119.8 ± 20.95	102.6 ± 12.18	80.62 ± 15.47
TGF-β	2,667 ± 81.38^***^	2,020 ± 134.6^**^^,^^#^	1,259 ± 256^###^^,^^††^	1,168 ± 8.36
IL-17	3,787 ± 89.68	6,687 ± 466.7	7,496 ± 645.5	7,015 ± 618.9
IFN-γ/IL-4 ratio	2.327 ± 0.42	2.91 ± 0.56	3.89 ± 0.36^**^	1.57 ± 0.25

*Results are presented as mean ± SEM*.

* *p < 0.05*,

** *p < 0.01*,

*** *p < 0.001 vs. dexamethasone (D) group*.

# *p < 0.05*,

## *p < 0.01*,

### *p < 0.001 vs. Z1 group*.

† *p < 0.05*,

†† *p < 0.01*,

††† *p < 0.001 vs. Z2 group*.

*Statistical comparisons were made using ANOVA with Tukey–Kramer multiple post-hoc test*.

In asthmatic animals treated with dexamethasone, all T cells subset (Th_1_, Th_2_, and Th_17_) were significantly decreased while Treg was increased compared to untreated asthma group (*p* < 0.001 for all cases; Figures 3A–D, 4A,C, 6A). Th_1_/Th_2_ ratio in the asthmatic group treated with dexamethasone was not significantly different with that of untreated group (Figure 6C).

The effects of treatment with all extract concentrations on Th_1_ and Treg were significantly higher than the effect of dexamethasone treatment (*p* < 0.01 to *p* < 0.001, Table 2). However, the effects of low and high extract concentrations on Th_17_, were lower than the effect of dexamethasone treatment (*p* < 0.01 and *p* < 0.05 for low and high extract concentrations, respectively; Table 2). In addition, the effect of high concentration of the extract on Th_1_/Th_2_ ratio was also significantly higher than that of dexamethasone (*p* < 0.001; Table 2).

### Cytokines gene expression

#### Cytokine gene expression in the spleen cells extracted from asthmatic animals

The gene expression of IFN-γ, FOXP_3_, and IFN-γ/IL-4 ratio were significantly reduced, while IL-4, TGF-β, and IL-17 gene expression were increased in asthmatic animals compared to the control group (*p* < 0.01 for IFN-γ and *p* < 0.001 for other cases, Figures 4B,D, 5B,C, 6B,D).

#### Cytokine gene expression in the spleen cells of treated groups with *Z. multiflora* extract and dexamethasone

Gene expression of IFN-γ in spleen cells of asthma groups treated with medium and high concentrations of the extracts (400 and 800 μg/ml; *p* < 0.05 for both cases) and that of FOXP_3_ in the group treated with medium extract concentration (*p* < 0.001) were significantly increased compared to untreated asthma group (Figures 4B, 5B).

Gene expression of IL-4 in the group treated with high concentration of the extract (*p* < 0.05), IL-17 in asthma groups treated with all extract concentrations (*p* < 0.001 for all cases) and TGF-β in the groups treated with the medium and high extract concentrations (*p* < 0.001 for both cases) were significantly decreased compared with untreated asthma group (Figures 4D, 5B, 6B).

The ration of IFN-γ/IL-4 in the group treated with high concentration of the extract was significantly increased compared with the untreated asthma group (*p* < 0.01; Figure 6D).

There was no significant difference in genes expression of IFN-γ, IL-4, IL-17, and IFN-γ/IL-4 ratio among the asthma groups treated with different concentrations of extract. The effect of the treatment with medium concentration of the extract (400 μg/ml) on FOXP3 gene expression was significantly higher than the other two concentrations (*p* < 0.001 for both cases). The effect of treatment with medium and high concentrations of the extract on TGF-β gene expression was significantly higher than treatment with its low concentration (*p* < 0.05 and *p* < 0.001 for the medium and low concentration, respectively). In addition, the effect of high concentration of the extract was higher than its medium concentration on TGF-β gene expression (*p* < 0.01, Table 2).

In animals treated with dexamethasone, gene expression of IL-4 (*p* < 0.05), IL-17 and TGF-β (*p* < 0.001 for both cases) were significantly reduced compared with untreated asthma group (Figures 4D, 5B, 6C). However, there were no significant changes in IFN-γ, FOXP3, and IFN-γ/IL-4 ratio in asthma group treated with dexamethasone compared with untreated asthma group (Figures 4B, 5B, 6D).

The effects of all extract concentrations on genes expression of IFN-γ and its medium concentration (400 μg/ml) on FOXP3 (*p* < 0.01 to *p* < 0.001) were significantly greater than the effect of dexamethasone. However, the effects of treatment with low concentration of the extract (400 μg/ml) on IL-4 gene expression and its two lower concentrations (200 and 400 μg/ml) on TGF-β gene, were significantly lower than the effect of dexamethasone (*p* < 0.05 to *p* < 0.001; Table 2).

## Discussion

The most important characteristic feature of asthma is chronic airway inflammation which is associated with increased number of Th_2_lymphocytes and their cytokines (IL-4, IL-5, IL-9, and IL-13) while Th_1_ lymphocytes and their cytokines (IL-2, IFN-γ, and IL-12) are reduced. There are also increased activated eosinophils and mast cells counts in asthma (Ray and Cohn, 1999; Larché et al., 2003). Th_2_ cells can induce B cell response to produce IgE and consequently activate mastocytes and eosinophils and regulate pro-inflammatory response, whereas Th_1_ cells directly or indirectly, inhibit inflammatory activities by preventing the Th_2_ response and thus, regulate the inflammatory responses of mast cells and eosinophils which are the main player cells in allergic asthma. Stimulation of Th_1_ may inhibit Th_2_ cells and allergic inflammation by secreting IL-2 and IFN-γ. IFN-γ not only suppresses Th2 responses but also stimulates B cells to secret IgG. Therefore, inducing an increase in Th_1_ activity could be considered as a treatment strategy for asthma (Lindemann and Racké, 2003). FOXP_3_ is essential for the differentiation of primary T cells to Treg phenotype. Treg cells control not only pathogenic Th_2_ cells, but also its effect on Th_1_ cells and the reduction of FOXP3 had been demonstrated in patients with stable asthma (Provoost et al., 2009). IL-17 may play an important role in the development of autoimmune disorders and chronic inflammation by secretion of other cytokines and growth factors from stromal cells (Molet et al., 2001; Annunziato et al., 2008). In addition, the effect of TGF-β during the late phase of lung inflammation and its role in airway remodeling in asthma has been reported (Magnan et al., 1996; Duvernelle et al., 2003). Asthma is an inflammatory disease and there is greater emphasis on its prevention using anti-inflammatory drugs in the treatment of this disease. Therefore, in this study, the preventive effect of *Z. multiflora* on Th cell sub types and the gene expression of several pro-inflammatory and anti-inflammatory cytokines in splenocytes of asthmatic BALB/c mice were evaluated.

In asthmatic animals, flow cytometry results showed significant increase in Th_2_ and Th_17_ but significant decrease in Th_1_ compared to control group. In addition, the ratio of Th_1_/Th_2_ in asthmatic animals was significantly decreased compared to control group.

IFN-γ and FOXP_3_ gene expression showed a significant decrease, while a significant increase was observed in IL-4, TGF-β, and IL-17 gene expression in spleen cells of asthmatic animals compared to control group. The ratio of IFN-γ/IL-4 in asthmatic animals compared to control group was also significantly reduced. These results confirmed the induction of experimental model of asthma in mice (sensitization of the animals) as previously shown using similar method of sensitization (Larché et al., 2003).

Th_2_ and Th_17_ cells in treated animals with three concentrations of the extract were significantly reduced but Treg cells was significantly increased compared to untreated asthma group. In addition, treatment with high concentration of the extract significantly increased Th_1_/Th_2_ ratio. The findings also showed that the effect of high concentration of the extract on Th_2_ cells and Th_1_/Th_2_ ratio was significantly higher compared to its low concentration. These results suggest a preventive effect for the plant on subset of T lymphocyte and gene expression of their cytokines in an animal model of asthma (sensitized animals) when the extract was administered during the sensitization period.

Although, the percentage of Th_17_ cells in Z1 treated group was higher than Z2 and Z3 treated groups, the differences between three concentrations of the extract on Th_17_ cells were not statistically significant. IL-17 gene expression also was not significantly different among treated groups with three different concentrations of the extract. In addition, IL-17 gene expression was significantly reduced due to treatment with all three concentrations of the extract which was consistent with the results of the extract on Th_17_ cells.

In previous studies, the relaxant effect of *Z. multiflora* and carvacrol as its main constituent on tracheal smooth muscle had been shown which may indicate the bronchodilator properties of the plant and carvacrol (Boskabady and Jalali, 2013; Boskabady et al., 2014a). The effect of *Z. multiflora* on cytokines of human lymphocytes and lung lavage of guinea pigs model of asthma showed reduced IL-4 and enhanced IFN-γ gene expression as well as increased IFN-γ/IL-4 ratio (Boskabady et al., 2013) which support the findings of the present study. In addition, *Z. multiflora* improved IL-8 level, total WBC, eosinophil counts, and MDA level in an experimental animal model of COPD (Boskabady et al., 2014b). In animal model of COPD, *Z. multiflora* extract and its constituent, carvacrol showed preventive effect on tracheal responsiveness and pathological changes of the lung that may be due to its anti-inflammatory properties (Boskabady and Mahtaj, 2015; Gholami Mahtaj et al., 2015). The effects of the plant on animal model of COPD also suggest the preventive effect of the plant on inflammatory lung disorders and support the findings of the present study.

Treatment of sensitized animals with dexamethasone caused a significant reduction in Th_1_, Th_2_, and Th_17_ cells and a significant increase in Treg cells compared to untreated asthmatic animals. The results of previous studies also showed that dexamethasone treatment lead to reduction of both IFN-γ and IL-4 gene expression in human lymphocyte and sensitized guinea pigs (Boskabady et al., 2013). The results of this study also showed that treatment of asthmatic mice with dexamethasone reduced Th_1_ cells and did not affect Th_1_/Th_2_ ratio, IFN-γ gene expression and IFN-γ/IL-4 ratio which was in line with the previous findings (Boskabady et al., 2013). The effect of treatment with the extract on Th_1_ and Treg cells was significantly higher and more selective than that of dexamethasone. The ratio of Th_1_/Th_2_ in asthmatic animals treated with the extract was also significantly higher than in treated with dexamethasone. The results of the present study showed that treatment of asthmatic animals with the extracts of *Z. multiflora* reduced Th_17_ and Th2 cells as well as IL-17, IL-4, and TGF-β gene expression but increased Treg cells as well as FOXP_3_ and IFN-γ gene expression. These results indicate the effect of the extract on both measured pro-inflammatory and anti-inflammatory cytokines. However, dexamethasone only affected Th_2_, Th_17_, and Treg subtypes and gene expression of only pro-inflammatory cytokines. Therefore, the findings of the present study suggested a more specific preventive effect for the plant in inflammatory disorders such as asthma compared to dexamethasone.

The extract also showed a dose dependently inhibitory effect on TGF-β gene expression. Treatment of animals with high and medium concentrations of the extract showed overexpression of anti-inflammatory cytokine (IFN-γ) in asthma and increased number of Treg (FOXP_3_) compared to untreated asthmatic animals. These findings may indicate more pronounced preventive effect of high and medium concentrations of extract on anti-inflammatory Th subsets.

Reduced IL-4, increased IFN-γ and enhanced IFN-γ/IL-4 ratio due to *Z. multiflora* extract were shown in OVA-sensitized guinea pigs as well as in PHA-stimulated human lymphocytes, using ELISA assay, previously (Boskabady et al., 2013) which support the results of the present study. However, it would be also helpful to examine the effect of the plant on the levels of cytokine using Western Blot and immunohistochemistry (IHC) in sensitized animals in future studies.

Taken together, these results may predicate the therapeutic effect of *Z. multiflora* extract on inflammatory diseases and immunologic disorders of Type I hypersensitivities (IgE-mediated) such as hay fever, urticaria, and asthma. The prophylactic effect of the plant extract on tracheal response, inflammatory mediators (Boskabady et al., 2014a) and pathological changes (Boskabady et al., 2014b) in sensitized guinea pigs.

In the present study the cellular and molecular mechanisms of the preventive effect of *Z. multiflora* on asthma was studied by flow cytometry measurements of Th subtype cells and also gene expression of their major cytokines which showed promising cellular and molecular effect of the plant on asthma. The results of the present study are in line and supported with the findings of previous studies of the effect of *Z. multiflora* on animal model of asthma and COPD (described in the rest of discussion previously). However, as it is clear all aspects of cellular and molecular bases of the effect of the plant could not be conducted in a single study and further studies needed to be performed in this regard in the future.

In conclusion, the results of the present study indicated that the extract of *Z. multiflora* decreased pro-inflammatory cytokines in asthma (IL-4, IL-17, and TGF-β) whereas increased anti-inflammatory cytokine (IFN-γ) gene expression in splenocytes of experimentally-induced asthma in mice. These results may indicate a more specific preventive effect for the plant extract compared to dexamethasone in allergy, autoimmunity and infectious diseases by both potentiating the production of Th_1_ cytokines and suppressing the inflammatory cytokines from Th_2_ and Th_17_. In future studies, Western Blot and immunohistochemistry should be conducted to ensure the effect of *Z. multiflora* on Th_1_/Th_2_ and Th_17_/T regulatory in a mouse model of allergic asthma.

## Author contributions

MB, MK, and AR designed the work, and analyzed and interpreted of data. MK, DH, and RN helped in execution of research. AA and MB wrote the manuscript and all the other authors read, improved and approved the manuscript.

### Conflict of interest statement

The authors declare that the research was conducted in the absence of any commercial or financial relationships that could be construed as a potential conflict of interest.

## Abbreviations

*Z. multiflora*: *Zataria multiflora*
BHR: bronchial hyper-responsiveness
OVA: ovalbumin
i.p: intraperitoneal
DTH: delayed-type hypersensitivity
Treg: regulatory T cells
β2M: β2-Microglobin
IFN-γ: interferon gamma
IL-4: interleukin 4
IL-17: interleukin 17
FOXP_3_: forkhead box P3
TGF-β: transforming growth factor beta
IgE: immunoglobulin E.
